# Supplementary material for: HMMER Cut-off Threshold Tool (HMMERCTTER): Supervised classification of superfamily protein sequences with a reliable cut-off threshold
Source: PLoS One. 2018 Mar 26;13(3):e0193757. doi: 10.1371/journal.pone.0193757 (PMC5868777; doi:10.1371/journal.pone.0193757)
Supplement: S1 Appendix — (PDF) [file pone.0193757.s001.pdf]

S1 Appendix HMMERCTTER procedure

Figure 1 outlines the HMMERCTTER training procedure as well as the target sequence analysis.

Dataset Preparation

The input consists of 1) A fasta formatted file containing the training sequences; 2) A Newick formatted tree of the corresponding sequences; 3) A fasta formatted file containing the target sequences. It is recommended the sequence annotations consist of up to 10 alphanumerical symbols only, in order to prevent communication errors between the various dependencies. In addition, annotations of training sequences should correspond 100% with the annotation of the tree. It is recommended to provide a target dataset that only contains homologs as further explained in the main text.

Training

The training process, based on the clustering of the training set, starts with the following input: 1) the training sequences; 2) the phylogenetic tree of the training sequences; 3) a minimum cluster size; and 4) the location of the output folder. The tree is read and midpoint rooted using Dendroscope [35]. Next, all monophyletic clusters with minimal cluster size are identified and sorted by descending size, resulting in a ranked list of clusters and a corresponding set of files containing the annotation codes for the sequences of each cluster.

In the first iteration the sequences from the highest ranked group (i.e. the largest monophyletic group) are retrieved from the training set, using a fetching script written in PERL. The sequences are aligned using MAFFT [34] with the option *–anysymbol –auto.* The resulting alignment is used to generate a HMMER profile using *hmmbuild* with standard settings. Then the cluster sequence set and the complete training set are

screened by *hmmsearch option –noali*, and finally the total scores are analyzed to determine if the cluster shows 100% P&R self detection. A 100% P&R self detection cluster is one whose HMMER profile identifies cluster members with a higher score than non-cluster members. If the cluster is 100% P&R it is presented to the user, as shown in S Fig 1A, who either accepts or rejects the cluster. Upon acceptation the following happens: 1) the lowest cluster member score is accepted as initial cut-off threshold for the cluster, which will be used later for the target or classification phase; 2) the cluster and all nested and partaking clusters are removed from the ranked list, since they will not be processed in the iterative process. In case of rejection, either for not being 100% P&R, or because of user decision, only the tested cluster is removed from the ranked list.

HMMERCTTER now iterates the above procedure with the next cluster on the the ranked list until it contains no more clusters. The last step of the training procedure is to annotate the tree using the standard color scheme of Dendroscope. The process output consists, besides a log file, of the following information: 1) The color annotated training tree in graphical and NEXML format; 2) A set of text files with annotation codes, corresponding to the selected 100% self detection P&R clusters; 3) a text file with the annotation codes of the orphan sequences.

Target Classification

The classification of the target data-set starts with the input of 1) the location of the training output folder that contains the input files for the classification; 2) the target sequences. The training and target datasets are combined into a unique dataset that contains all the sequences.

For each cluster, HMMER profiles and cut-off thresholds are computed based on their sequences, retrieved from the combined data set, similarly as described in the training phase. All these initial profiles are based only on training sequences. A

hmmsearch is applied with all profiles over the combined data set, in order to classify the sequences that score above its threshold. All sequences, including the original training and target sequences, are provisionally accepted and the result is checked for classification conflicts. Two types of conflict may occur: 1) A training sequence is identified by another cluster or group; II) A target sequence is identified by more than one cluster or group. In both cases, the involved groups will no longer be iterated. Conflicting training sequences are removed from all except the group of the original training cluster. Conflicting target sequences are removed from all groups as well as the complete dataset. Also, provisionally accepted sequences with scores lower than a conflicting sequence, are not accepted and removed from the complete dataset. All other sequences are accepted. Note that in the automated classification step sequences are accepted based on information that does not include the information of the accepted sequences themselves (prior sequence inclusion). Upon acceptance and inclusion the sequences alignment, profile and threshold are updated for the next iteration.

The above procedure is iterated until no more positives are detected or all groups have been marked as conflicting. This results in a new group of 100% P&R self detection groups, which include training and target sequences, with group-specific HMMER profiles and thresholds based on all the group's sequences.

The automated procedure is followed by an interactive procedure in which seemingly negatives can become included based on a posterior, rather than prior, inclusion test of 100% P&R self detection. Guided by an interface such as shown in S Fig 1B, the user can select one or ten sequences, set the cut-off threshold manually or use the largest score-drop, below the current threshold, to define a new cut-off threshold. The sequences with scores above the new threshold are included, novel MSA and HMMER profiles are made and used to test if these sequences classify into the group upon

inclusion (score above threshold). If 100% P&R self detection is lost, the user is notified and needs either to accept the former group as it was before, or to include less novel sequences. When 100% P&R self detection is attained, two scenarios exist. 1) An updated cluster identified novel sequences since it has a more sensitive classifier. The user is notified and should accept or reject an automated inclusion of these positives, according to what is described above. 2) No novel positives are detected and the updated group is presented to the user, whom can add more sequences but also reject the latest addition since maintaining 100% P&R self detection can still result in a severe deterioration of the quality of the group The latter can be identified by the analysis of the score plot that is presented to the user by the software in each iteration (S Fig 1B). When the user accepts a group (when no more sequences can be added without losing either 100% P&R or the significant score-drop) the next group is iteratively analyzed until there are no more groups to process. Output consists, besides a log file, of the following: 1) a set of text files with annotation codes, corresponding to the original clusters supplemented with those of the positively identified sequences; 2) a text file with the conflicting sequences; 3) All files (MSA, HMMER profile and HMMSEARCH output) for the latest iteration of each group.
