# Supplementary figures and images for: HMMER Cut-off Threshold Tool (HMMERCTTER): Supervised classification of superfamily protein sequences with a reliable cut-off threshold
Source: PLoS One. 2018 Mar 26;13(3):e0193757. doi: 10.1371/journal.pone.0193757 (PMC5868777; doi:10.1371/journal.pone.0193757)

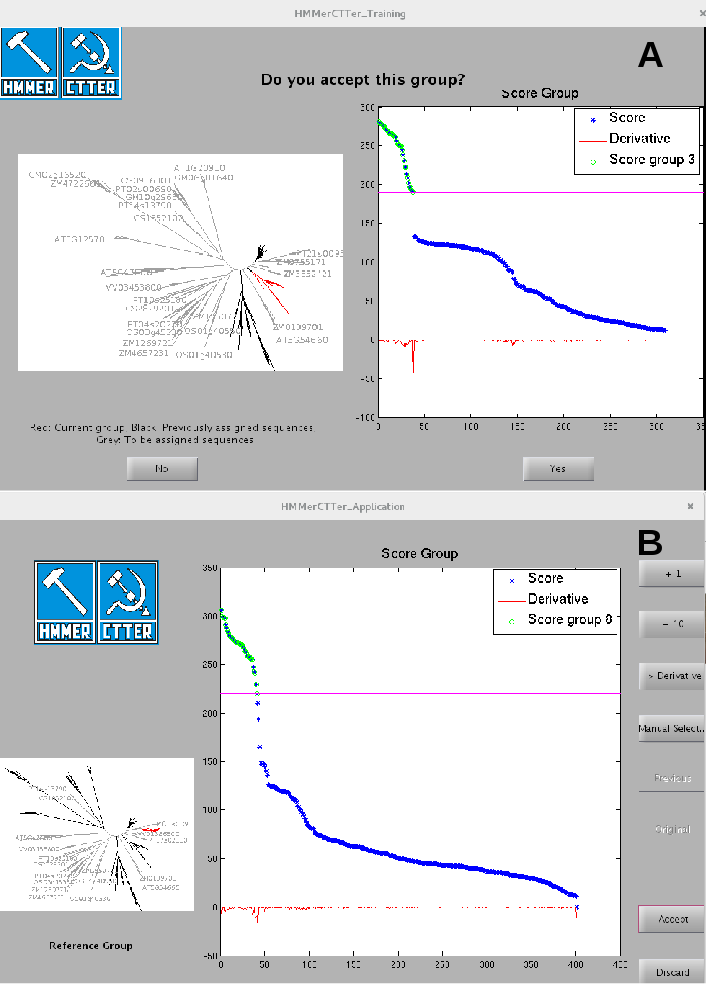

Supplement: S1 Fig — Shown are single examples obtained from the training (A) and the target phase (B). Blue asterisks are scores of sequences of the complete dataset (either training or combined), green circles are scores of sequences of the cluster or group in question. The red line shows the drop in scores among successive sequences, also indicated as score drop. The magenta line shows the current threshold and can be moved in order to determine the threshold in the interactive part of the classification. In the training the user must accept or reject the 100% P&R cluster. During classification the user can accept the cluster, return to the former or initial state or add seemingly negatives as indicated for posterior 100% P&R self detection testing. (TIF) [file pone.0193757.s002.tif]

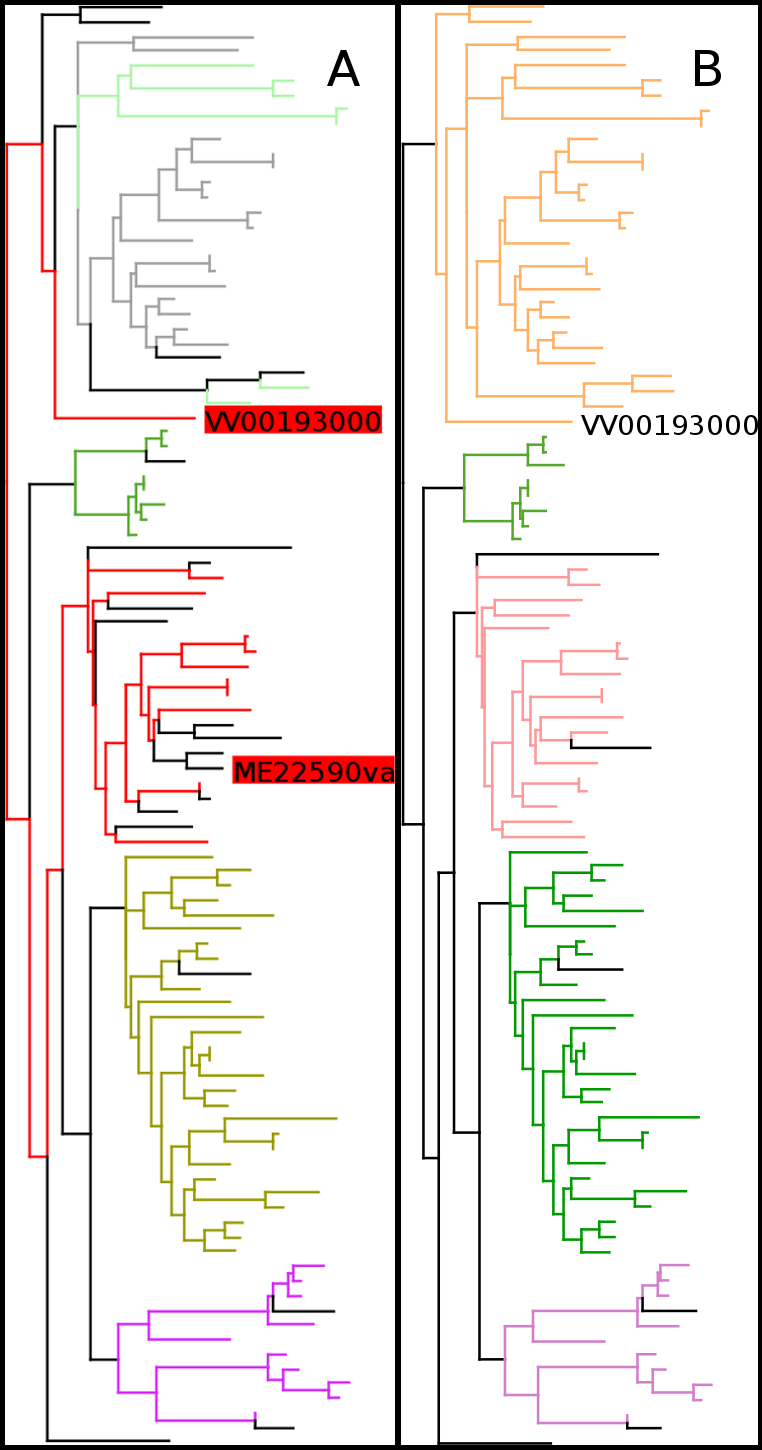

Supplement: S2 Fig — (A) Detail of poor classification of first run using clustering based on train-tree with sequence VV00193000 clustered differently than in the shown final tree. Sequences of GR1-11 including VV00193000 in red; GR1-15 in gray nested inside the clade containing GR1-22 light green lines. Target sequence ME22590va contains three ACDs also generating classification conflicts. (B) Detail of improved classification of second run obtained upon removal of VV00193000 and ME22590va from training and target set, respectively. GR2-14 shows 80% classification recall, formerly 0% with G1-11. GR2-9 shows 100% classification recall, which is an improvement of the 67 and 50 of constituents GR1-15 and GR1-22. Numbers also in Fig 1. (TIF) [file pone.0193757.s003.tif]

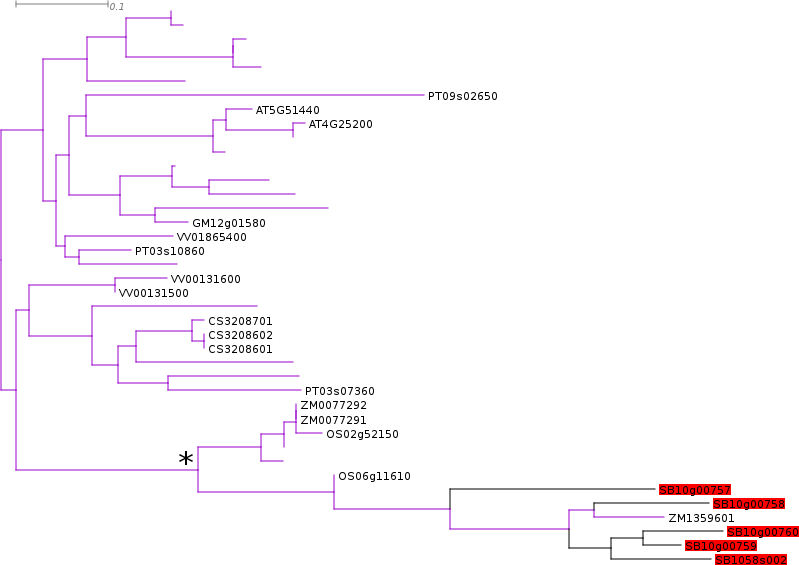

Supplement: S3 Fig — Detail of cluster 7/M+ demonstrating that particularly sequences at large distances (ML) are often not detected. The indicated sequences without shading are training sequences whereas the sequences in red shade are false negatives. The arrow points to a monocotyledon sub clade. (TIF) [file pone.0193757.s004.tif]

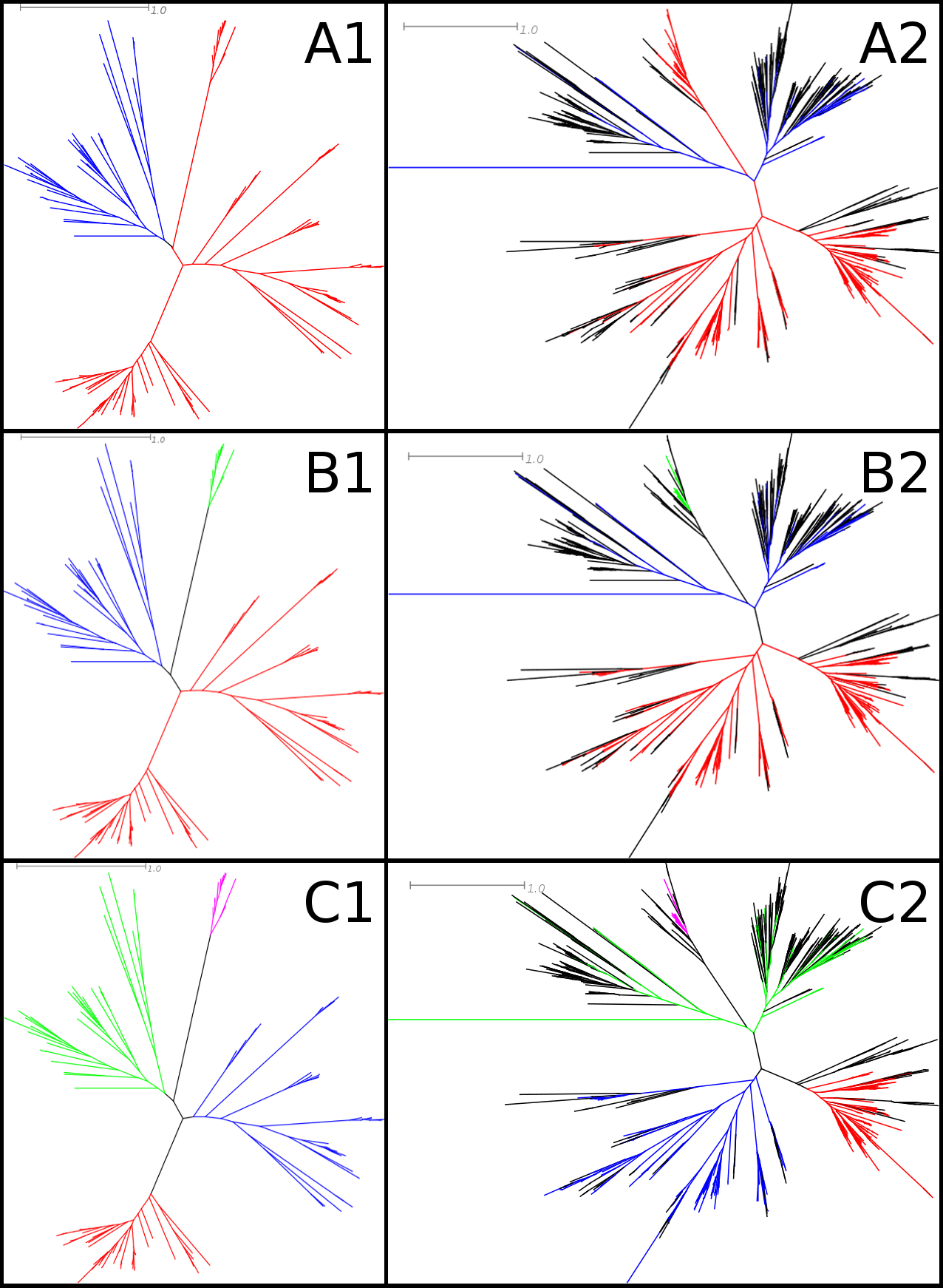

Supplement: S4 Fig — (A1) C2 Clustering; (A2) C2 Classification; (B1) C3 Clustering; (B2) C3 Classification; (C1) C4 Clustering; (C2) C4 Classification. Colors according to HMMERCTTER output, leaves in black could not be clustered or classified. Scale bars indicate 1 amino acid substitution per site. (TIF) [file pone.0193757.s005.tif]

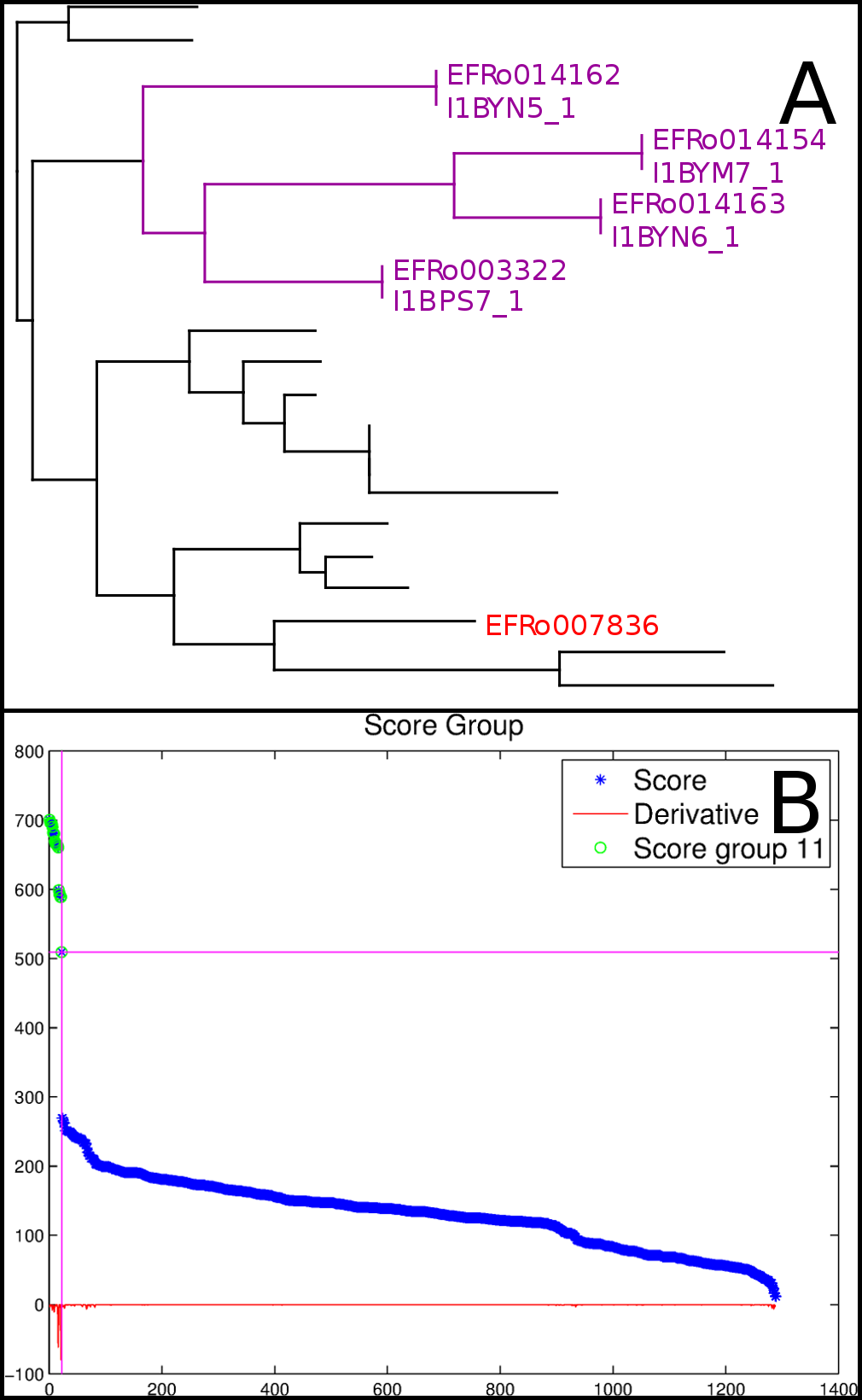

Supplement: S5 Fig — (A) Classification of group 7 by C7 clustering. In purple sequences from C7-G7 group. Other leaves represent sequences identified by the C11 clustering. EFro007836 is identified by both C7-C2 and C7-C7, preventing classification. (B) Score plot of C11-G7. Blue asterisks are scores of sequences of the complete dataset, green circles are scores of sequences group C11-G7. The red line shows the drop in scores among successive sequences. The magenta line shows the current threshold. (TIF) [file pone.0193757.s006.tif]

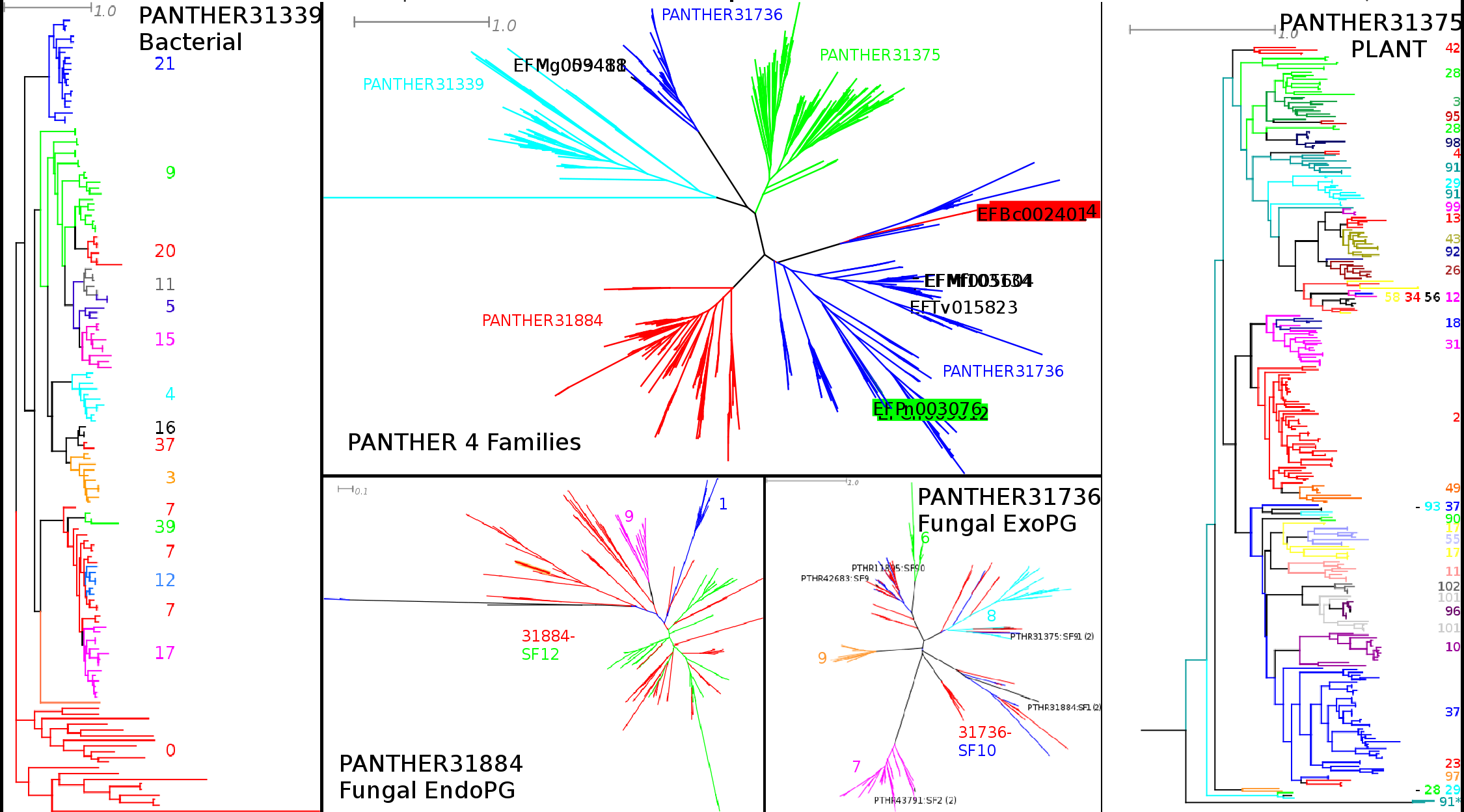

Supplement: S6 Fig — PANTHER classification was analyzed at combined family, as well as at individual subfamily level. “Panther 4 Families” shows a very good classification, false positives are indicated with colored labels, false negatives are indicated in black. The latter are indicated with their PANTHER identifier in the 31736 panel. The four families were analyzed at the subfamily level. Subfamily numbers are indicated in the same color as the corresponding edges,—indicates sequences correspond to a family but were not classified into a specific subfamily. (TIF) [file pone.0193757.s007.tif]

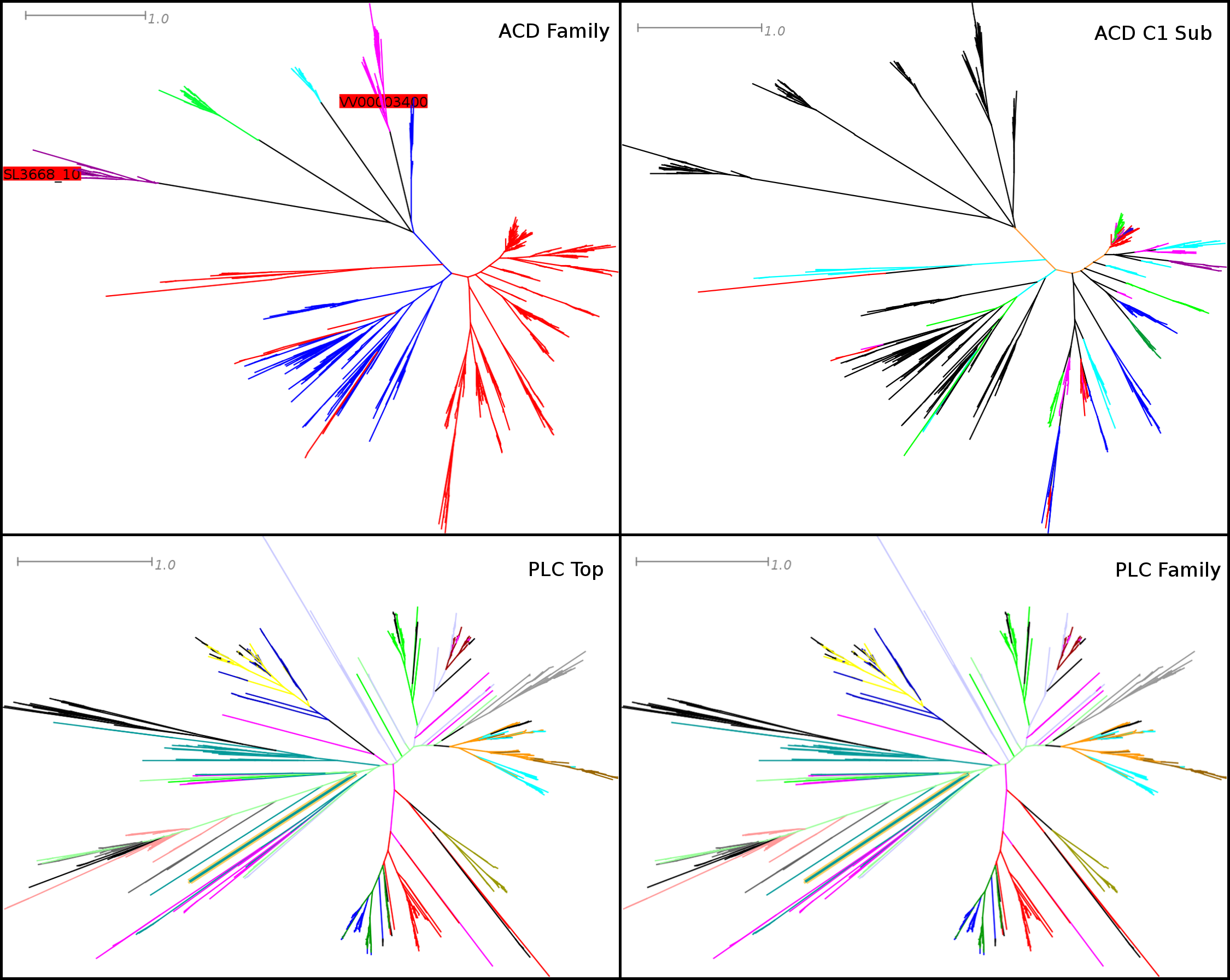

Supplement: S7 Fig — The top panels show the ACD family level and the sHSP subfamily level classifications. Each family is respresented by a different color. For the sHSP subfamily classification colors were chosen to represent separate clusters. The lower panels show the classification of all PLC subfamilies containing a training sequence as well as two additional large subfamilies (PLC Top, random colors) and the classification upon combining subfamilies according to the functional classification according to SwissProt (For colors see Fig 4A). Data analysis is shown in Table 1. (TIF) [file pone.0193757.s008.tif]
